# A Review of Data-Driven Approaches and Techniques for Fault Detection and Diagnosis in HVAC Systems

**DOI:** 10.3390/s23010001

**Published:** 2022-12-20

**Authors:** Iva Matetić, Ivan Štajduhar, Igor Wolf, Sandi Ljubic

**Affiliations:** 1 Faculty of Engineering, University of Rijeka, Vukovarska 58, HR-51000 Rijeka, Croatia; 2 Center for Artificial Intelligence and Cybersecurity, University of Rijeka, R. Matejcic 2, HR-51000 Rijeka, Croatia

**Keywords:** fault detection and diagnosis, HVAC systems, data-driven approach

## Abstract

Heating, ventilation, and air conditioning (HVAC) systems are a popular research topic because buildings’ energy is mostly used for heating and/or cooling. These systems heavily rely on sensory measurements and typically make an integral part of the smart building concept. As such, they require the implementation of fault detection and diagnosis (FDD) methodologies, which should assist users in maintaining comfort while consuming minimal energy. Despite the fact that FDD approaches are a well-researched subject, not just for improving the operation of HVAC systems but also for a wider range of systems in industrial processes, there is a lack of application in commercial buildings due to their complexity and low transferability. The aim of this review paper is to present and systematize cutting-edge FDD methodologies, encompassing approaches and special techniques that can be applied in HVAC systems, as well as to provide best-practice heuristics for researchers and solution developers in this domain. While the literature analysis targets the FDD perspective, the main focus is put on the data-driven approach, which covers commonly used models and data pre-processing techniques in the field. Data-driven techniques and FDD solutions based on them, which are most commonly used in recent HVAC research, form the backbone of our study, while alternative FDD approaches are also presented and classified to properly contextualize and round out the review.

## 1. Introduction

The optimization of energy efficiency in HVAC systems represents a challenging problem that researchers and engineers worldwide are trying to solve. The main issue with HVAC systems is that they frequently waste energy on suboptimal thermal comfort, which has a greater detrimental effect on the environment because the energy used in this context mostly comes from nonrenewable sources. The mentioned problem is further accentuated by growing human population and globalization. As the population grows, so does the desire for improved living conditions, which must be followed by established nations’ lifestyles and high energy consumption habits [1]. A statistical analysis and overview of heating and cooling systems and their energy consumption is given by International Energy Agency (IEA) [2,3,4]. Reports show the buildings being linked to 2.9 Gt of total $CO_{2}$ emissions in comparison to electricity (13.5 Gt), industry (8.5 Gt), transport (7.2 Gt), and other entities (1.9 Gt). Without significant efficiency improvements in cooling technology, worldwide power consumption for cooling in buildings might rise by up to 40% by 2030. Furthermore, heat pumps, which are regarded to be part of HVAC systems, are the fastest-growing heating technology, with a prediction of 20% influence on worldwide heat demand for buildings by 2030 [3]. In [5], energy consumption is observed in a typical office building. Results showed that HVAC system has a majority share of 39% in comparison to lighting (25%), equipment (22%), lifts (4%), domestic hot water (1%), and all other entities (9%). On a positive note, according to the IEA’s Net Zero Emissions Strategy [4], $CO_{2}$ emissions from the building category should be reduced by 40% between 2020 and 2030, by moving away from the use of fossil fuel boilers and upgrading the existing building stock to increase energy efficiency. Additionally, the IEA organization further plans to implement zero-carbon-ready buildings that are energy efficient through the use of renewable energy or an energy source that will be decarbonized by 2050 under the net zero plan.

HVAC systems operate under dynamic conditions and can therefore be unpredictable due to sudden disturbances and/or malfunctions. This requires to make buildings smarter, which can be achieved by fully exploiting sensor-based infrastructure and implementing FDD methods. A variety of factors make FDD in HVAC systems critical. Faults can result in low-quality indoor air, inadequate heating or cooling, or both. The health and well-being of the occupants can be negatively affected by these problems. Inefficient energy use leads to higher energy costs for building owners and operators, as well as higher energy bills. In addition, inefficient HVAC systems can cause equipment to wear out prematurely and require more frequent repairs, driving up maintenance costs. Another important issue is safety. Many hazardous materials, including fuels and refrigerants, are used in contemporary HVAC systems. Leaks, fires, and other safety hazards are possible within HVAC systems if they are not properly maintained and operated. Potential safety risks can be found and corrected before they occur with the help of FDD. Therefore, FDD is essential for detecting and correcting problems with HVAC systems in order to maintain their functionality and avoid these negative effects.

Implementation of FDD methods can be made through data-driven or hybrid approaches that leverage machine learning (ML). In today’s world, where big data are a common component of building energy management systems (BEMSs), ML is the best tool to further optimize HVAC systems, with the goal of monitoring energy efficiency, regulating thermal comfort, and reducing environmental footprint in the long run.

### 1.1. Recent FDD Methodology Reviews

There has been a great deal of interest in reviewing the field of HVAC FDD methods. Recent reviews present trends in the use of FDD methods, explain the methodology of each commonly used algorithm, and develop new ways to categorize FDD methods and techniques [1,6,7,8,9,10,11,12,13,14,15,16,17,18,19,20,21]. Li et al. [6] specifically reviewed FDD methods with a focus on feature engineering in FDD. Next, Hosseini Gourabpasi and Nik-Bakht [7] developed knowledge discovery models that inferred common researched faults and used features along with their connections to different parts of the HVAC system. Furthermore, Himeur et al. [8] developed and shared a systematic categorization of all anomaly detection schemes in energy consumption, accompanied by detailed descriptions. They also provided a detailed explanation of the domain-specific challenges and limitations of energy consumption fault detection. A review on data-driven approaches and data mining methods was presented in [9], wherein the authors examined the FDD context with a focus on air handling unit (AHU) systems and chillers. An overview of the implementation of FDD methods aimed at dynamic thermoeconomic and diagnosis analyses in HVAC systems was given by Picallo-Perez et al. [10]. In addition, a new categorization of FDD approaches has also been proposed in the same study. Yu et al. [11] studied FDD methods applied exclusively in residential air conditioning (AC) systems and focused their research on evaluating each method in terms of sensor requirements. They also tackled a subject of fault detection via smart thermostats.

Some existing reviews [12,13,14,15,16,17,18,22,23] specifically aim to categorize and compare different FDD methods in HVAC systems. Among them, the study by Katipamula and Brambley [17] should be highlighted, which had a significant impact on the development of other scientific publications. Namely, their detailed analysis of the categorization of FDD methods is often cited in other works.

### 1.2. Contribution and Review Structure

The aforementioned reviews lack the identification of systematic workflows that would help researchers and solution developers, whether new to the field or more experienced, deal with existing FDD approaches and methods more easily. Some of the reviews focus mainly on only one HVAC subsystem and ignore all other HVAC subsystems, which consequently leads to a limited perspective. While good work has been achieved in elaborating and comparing FDD techniques, what distinguishes this research from the existing ones is our holistic view from different perspectives based on common problems in HVAC systems as a whole. Through a comprehensive analysis of FDD methods in relation to all HVAC subsystems, we hope to achieve two goals:

- to help future researchers by providing them with a systematic overview of the state-of-the-art in HVAC FDD methods so that they can more quickly obtain all relevant information on this topic;
- to provide a set of heuristics (i.e., best-practice scenarios) for developers of advanced data-driven solutions in HVAC systems.

The above points define the specific contributions of this work.

As stated before, this review focuses on systematizing recent research in the HVAC FDD field, with an emphasis on data-driven approaches. Namely, data-driven techniques and FDD solutions based on them are the most prevalent in the HVAC studies reviewed and therefore deserve special attention. However, in addition to the most common data-driven solutions, we have also presented other alternative approaches and classified them accordingly to provide a comprehensive overview of the field. For example, we explain how these other approaches can also leverage data-driven FDD models, which is particularly important for the adoption of a hybrid approach. In this way, we were able to analyze data-driven techniques from many different perspectives.

The review is structured as follows. Section 2 details the utilized methodology for the systematic literature review. In particular, the database search procedure and the compilation of the most relevant papers from the selected time period are described. In Section 3, we present a new classification of FDD approaches, which is further elaborated with corresponding tables and references. Specifically, we present the literature on data-driven approaches from the point of view of labeled and unlabeled datasets, which is one of the main challenges in data-driven approaches. In Section 4, we focus on state-of-the-art techniques for data preprocessing and for developing data-driven models. In addition, we propose two workflows for selecting an appropriate FDD approach and deciding which model to use. This should help future researchers to find the FDD approach best suited for their work. Finally, in Section 5 we summarize our work and provide some concluding remarks on future research trends and possible obstacles to watch out for.

## 2. Methodology

**Figure 1:**
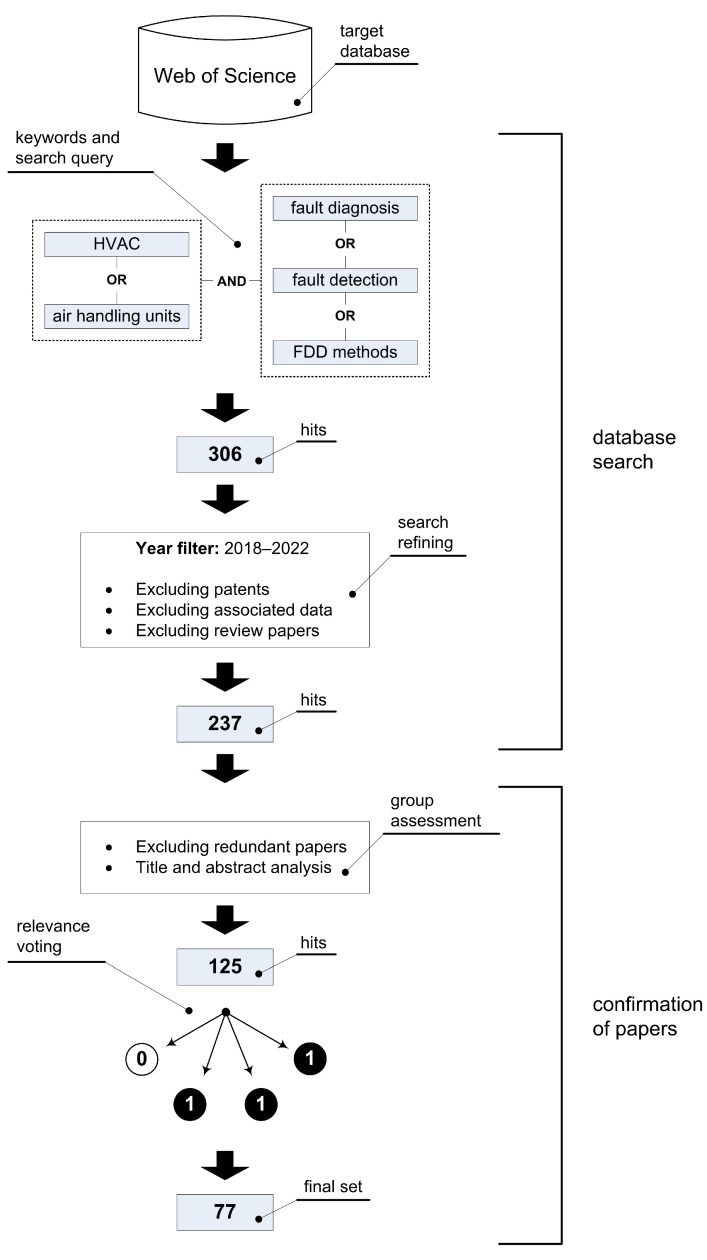
Workflow for collecting the literature.

First, we define the systematic literature review (SLR) methodology used in our study. The process of selecting recent high-quality research papers targeting the HVAC FDD methods is illustrated by a workflow shown in Figure 1. The corresponding procedure involves two main parts: (1) a database search, which includes the definition of keywords, key phrases, and search query, as well as the refinement (i.e., filtering) of the initial result set, and (2) a confirmation process, in which the remaining papers are analyzed in more detail and subjected to relevance voting. In the following text, the individual steps of this workflow are explained.

### 2.1. Database Search

Data were collected in July 2022 via the Web of Science (WoS) platform, a widely used and authoritative database of research publications and citations. Since FDD is a specific niche in the HVAC industry, our search query consisted of two main components. The main keyword was obviously ‘HVAC’, but we decided to additionally include ‘Air handling units’ at this level. By including this most commonly used HVAC subsystem in the literature search, we wanted to obtain a result set that covers all methods commonly used in this field. The second part of the query consisted of different forms of FDD, such as ‘FDD methods’, ‘fault detection’, and ‘fault diagnosis’. After defining the mentioned keywords and key phrases, they are combined into a formal query represented by the logical expression (1), where the abbreviations and symbols indicate the following:

- *Q*—Search query
- *H*—‘HVAC’
- AHU—‘Air handling units’
- FDE—‘Fault detection’
- FDI—‘Fault diagnosis’
- FDDM—‘FDD methods’
- ∧—operator AND
- ∨—operator OR

(1) $$\begin{array}{c} Q=(H\vee AHU)\wedge (FDE\vee FDI\vee FDDM) \end{array}$$

Additional year filters were added so that we could search only from 2018 to July 2022. The total number of results was 306, which was then further refined by excluding review articles, patents, related data, and redundant papers, yielding a total of 237 hits.

### 2.2. Confirmation of Papers

After excluding the redundant papers, high-level relevance assessment of the research papers were performed by grouping all papers for each author to analyze the titles and abstracts in more detail. If no descriptions, claims, or results were found in the abstract that were important for inclusion in a comprehensive systematization, then the corresponding paper was not further considered. After this analysis, the number of literature papers found was 125.

Next was the low-level relevance check, the most time-consuming step, which involved a thorough inspection of all remaining papers and their group assessment (i.e., relevance voting). Namely, after reading the papers, each author decided for themselves whether something should be included or excluded from further study by giving the literature in question a 1 for inclusion or a 0 for exclusion. If 3 (or more) of 4 authors were in favor of excluding a paper, it was excluded. The decision criteria we used in this step to include a particular paper can be summarized as follows:

- The FDD-based problem in the HVAC domain is clearly stated;
- A solution is presented that is related to a real-life HVAC system or a corresponding simulation model;
- The techniques used are described consistently, unambiguously, and not vaguely;
- If data-driven techniques are used, the dataset should be explained in detail (i.e., data types, dimensionality, sampling rate, and other properties should be specified);
- Results obtained are contextualized/compared to related work;
- Conclusions can help to achieve the research goals (classify FDD approaches in HVAC systems and provide heuristic guidelines).

It is important to emphasize here that the authors conducted the above assessments in parallel and completely independently to minimize the risk of misunderstanding, i.e., to make the final selection of papers without bias. When disagreements arose about a particular paper, no additional arbitration was performed, as a simple majority rule was used to exclude the paper in question.

After this step, a final set of 77 representative papers from the target field remained. This set constitutes the body of knowledge on which this work is based.

### 2.3. Literature Statistics

**Figure 2:**
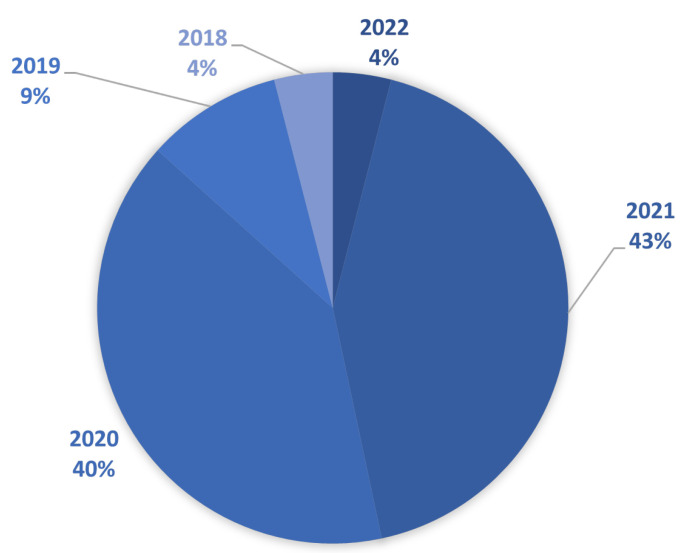
Overview of gathered literature aggregated by the publication year.

**Figure 3:**
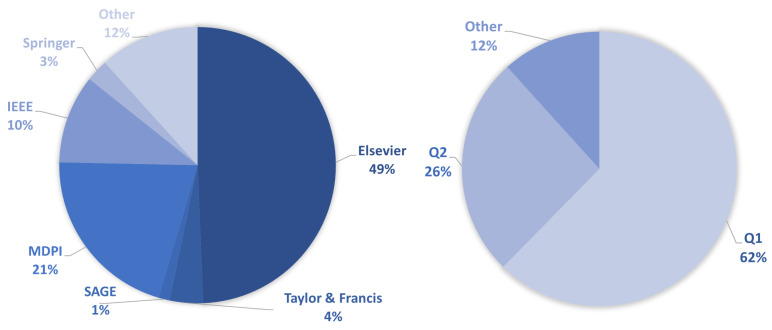
Overview of gathered literature concerning both the publishing organization and quartile-based ranking of the corresponding journal.

**Figure 4:**
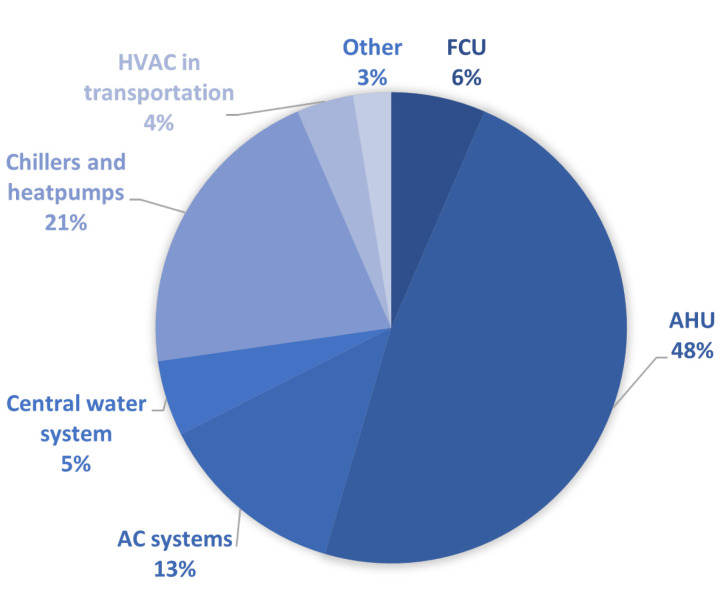
Overview of gathered literature concerning the analyzed HVAC (sub)system type.

The basic statistical data of the finally selected literature are presented in Figure 2, Figure 3 and Figure 4. Figure 2 shows a graphical interpretation of the entire dataset, filtered by year of publication. It can be seen that most of the selected papers were published in 2021 (43%) and 2020 (40%).

Figure 3 (left) shows the sources of the selected research literature. In terms of journal publishers, most of the selected articles come from Elsevier (49%), followed by MDPI (21%), IEEE (10%), Taylor & Francis (4%), Springer (3%), and SAGE (1%). Other sources (12%), include titles from conference proceedings published by IEEE, IBPSA (International Building Performance Simulation Association) and E3S Web of Conferences. In the same figure, on the right, we can see that the target work is of high quality. Namely, most of the selected papers are from journals belonging to quartile Q1 (62%), followed by quartile Q2 (26%) and the Other category (12%), which includes conference papers with undefined ranks.

Finally, in Figure 4, the selected literature was grouped by the respective HVAC subsystem. The selected papers address air handling units (AHU) (48%), chillers and heat pumps (21%), air conditioning systems (AC) (13%), fan coil units (FCU) (6%), central water systems (5%), HVAC systems in transportation (4%), and other HVAC categories (3%). The AC group furthermore includes rooftop units (RTU), variable refrigerant flow/volume (VRF/VRV) systems, refrigeration compressor rack units (RCU), multisplit systems, and packaged air conditioning units. A central water system includes central heating and cooling systems.

In categorizing the literature that falls under AHUs or chillers and heat pumps, we found that there are different viewpoints in the literature. For example, when fault detection was studied from the point of view of air-side faults (airflow, temperature, humidity), these papers were categorized as AHU-based. On the other hand, if the fault observation was from the energy consumption side, then this work fell under the investigation of the operation of the unit, such as a heat pump or chiller. The three most commonly studied areas are ventilation systems, chillers and heat pumps, and air conditioning systems. The pie chart in Figure 4 shows that other subsystems such as central water systems, fan coil units, or HVAC equipment in transportation are not represented to such an extent.

## 3. Results Part I: Review and New Classification of FDD Approaches in HVAC Systems

When we began to review the FDD-related literature, we found that there is no universally accepted classification for FDD approaches. Based on several existing articles [7,9,11,12,13,14,15,16,17], various classification strategies have been proposed, such as the following:

- qualitative-based, quantitative-based, and process-history-based models [17];
- data-driven and knowledge-driven approaches [13];
- model-based and data-based methods [16];
- black-box, gray-box, and prior knowledge-based models [15].

**Figure 5:**
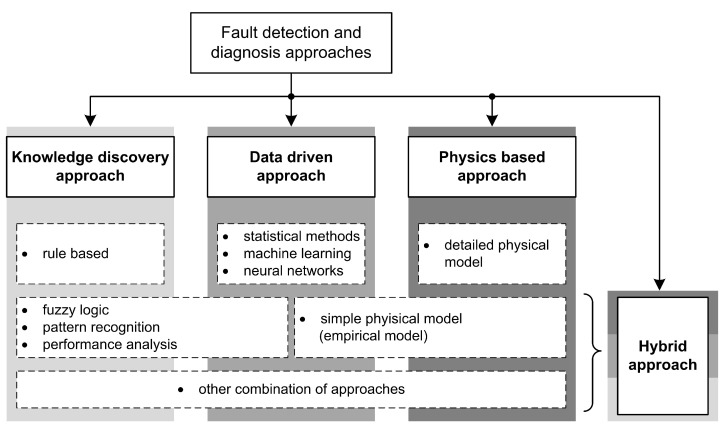
The proposed classification of FDD approaches in HVAC systems.

In this paper, we propose a new way to classify FDD approaches because existing work is either entangled or lacks systematic decomposition; therefore, it needs to be improved by extending and appropriately (sub)dividing FDD concepts. Our proposal considers the classifications from the existing work and combines and rearranges them accordingly. In this way, we capture the main idea behind each approach. In our classification, we distinguish the four main approaches: (1) knowledge discovery approach, (2) data-driven approach, (3) physics-based approach, and (4) hybrid approach. The proposed categorization is visualized in Figure 5.

**Figure 6:**
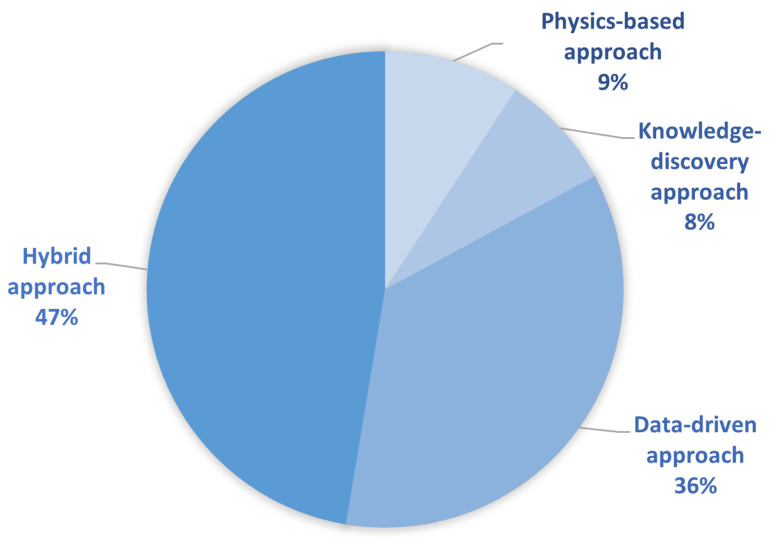
Overview of the selected literature grouped according to the proposed FDD classification.

The papers we collected during the selection process are distributed according to the proposed FDD classification as shown in Figure 6. In the following subsections, each category is explained separately, and an overview of the selected literature on each approach is given. Since the most commonly used approaches are data-driven or hybrid ones, and hybrid approach usually involves some form of data-driven techniques, we place the data-driven context at the center of our research.

In this review, the approaches, techniques, algorithms, and models analyzed are often presented with their corresponding acronyms, some of which are introduced for the first time in the text itself and others in accompanying tables. In this context, we draw the reader’s attention to the fact that a list of all abbreviations can be found at the very end of the paper, both to facilitate navigation and to improve readability.

### 3.1. Physics-Based Approach

In the literature, there have often been conflicts in classification of this approach. Namely, the physics-based model can be considered a white-box model [24], but some attribute it as a gray-box model [9]. To resolve this categorization conflict, we propose a separation between simple and complex physical models, where the simple physical model would belong to the hybrid approach. A detailed physical model is essentially a complex model that requires a lot of information about the building’s properties in order to model it correctly with a simulation software. However, when such a model becomes computationally too intensive to run, data-driven techniques are used to augment it and further optimize its performance. In this way, a simplified physical model, also called an empirical model, is created. Although it is based on the physics properties, we can classify it as a gray-box approach or a hybrid approach. Further details on the simple physics-based approach are given in Section 3.4.

When properly developed, physical models provide high accuracy, but can be difficult to handle because extensive data on the physical principles governing the thermodynamic processes must be collected and analyzed, and a large number of parameters must be incorporated in the model.

As shown in Table 1, the authors who have used the physics-based approach for FDD in HVAC systems have focused their research on developing simulations using specialized software [25,26,27,28,29,30]. In particular, detailed physical white-box models were analyzed in [25,26,27]. For example, Li et al. [30] conducted a comparative study of three different HVAC subsystems with EnergyPlus simulations based on the same modeled physical building. In each simulation, they created a sensor error offset and analyzed the impact on the ground source heat pump, variable refrigerant flow, and chiller-and-boiler system. They concluded that the heat pump system was the least affected by the sensor offset in terms of indoor comfort, energy consumption, and operating efficiency, while the chiller-and-boiler system was the most sensitive of the three systems observed. Next, Rosato et al. [25] performed detailed laboratory simulations in the Trnsys software, which were then tested on a case study of an office building. They tested various soft errors in the simulations to see how they could improve the performance of the AHU system. Their model is able to predict FCU behavior and the effects of faults on FCU performance, as well as to associate symptoms with faults under different control strategies. Shi and Augenbroe [26] attempted to develop a simulation that could serve as an evaluation tool for data-driven algorithms. Others focused more on creating simplified physical models, such as a novel bilinear model [29] and a RTU performance curves model [28]. Finally, a mathematical estimation model for sensor FDD was developed using an exponential function that locates the faulty sensor and removes noise in the data. The model was tested by simulations, but the underlying software was not mentioned [31].

### 3.2. Knowledge Discovery Approach

The knowledge discovery approach refers to a white-box model that relies heavily on expert knowledge. The approach is subject to the same limitations as the physics-based approach. However, when a complete mathematical model is not available and data are limited, this perspective is more advantageous. It is also easier to integrate into complex HVAC systems.

If-then rules are the best and most commonly used representation for implementing this approach. Table 2 lists examples from selected literature that address the knowledge discovery approach, all of which utilize if-then rules [32,33,34,35,36,37].

For example, Chen et al. [35] have developed a unified taxonomy for HVAC faults. It helps to classify equipment faults according to their characteristics and casual relationships. Furthermore, Lei et al. [33] have proposed a formalized fault definition for control logic with ontological reasoning for AHUs. This formalized control logic has the advantage of avoiding human interpretations and generalizing errors, making this method applicable to any system. The main objective of the research conducted by Woradechjumroen and Leephakpreeda [37] was to improve FDD analysis by implementing two steps: (1) reducing the outliers caused by the effects of adverse interaction between HVAC and RCU operation, and (2) isolating the effects of adverse interaction from the overall operation of refrigeration systems. The proposed method improves the data reliability and robustness of FDD. On the other hand, Nehasil et al. [32] combined rule-based fault detection with semantic data descriptions and cloud architecture with the goal of creating a general-purpose FDD system. Lin et al. [34] developed and implemented nine algorithms for error correction for HVAC systems. When triggered, the algorithms essentially override the control setpoints and other variables to affect the system. Finally, Deshmukh et al. [36] developed a fault detection algorithm that detects generic faults so that it is transferable and can be extended to more complicated systems. The algorithm looks for faults in historical data and is not predictive in nature.

### 3.3. Data-Driven Approach

The data-driven approach, also known as the process-historical, refers to black-box modeling and is based solely on sensory measurement data, without assigning any specific physical meaning to the measurements, and does not take into account the characteristics of the building in question [38,39,40]. Its primary objective is to mathematically link observed inputs to measurable outcomes. Moreover, no expert knowledge or understanding of the physical aspects of HVAC systems is required, which greatly simplifies model development. In essence, these models are flexible to the changing system behavior of the building and can be easily scaled to meet system needs [41]. On the other hand, these models often require large amounts of historical data and a wide range of sensor inputs that are not readily available in all buildings. In this regard, the costs associated with the quality and variety of sensors utilized to measure the data, as well as the time required to obtain a sufficient amount of historical data–both of which are essential to building a data-driven model—can be overwhelming [15].

An overview of related work addressing the data-driven approach in HVAC FDD is presented in Table 3.

When working with measurement data from HVAC systems, a common problem is that the data are unlabeled and have anomalies. In particular, multivariate, non-linear, and unpredictable time series data with recurring anomalies require extensive expertise in this area to label specific events as meaningful faults and errors. This can lead to limited solutions, as faults in HVAC systems can only be detected and resolved by experts in the field. On the other hand, quite a few datasets have been provided by the American Society of Heating, Refrigerating and Air-Conditioning Engineers (ASHRAE) [78] specifically for this field. As a result, the ASHRAE dataset is one of the most commonly used datasets for HVAC FDD methods, whether for validating a model or for training the model. Apart from special datasets labeled by experts, there are still no solutions for automatic detection of errors in an unlabeled dataset.

Given the availability of annotated datasets, the data-driven approach (as well as the hybrid approach that incorporates some data-driven techniques) can be categorized by type of learning as follows:

- Supervised learning
- Unsupervised learning
- Semi-supervised learning

It should be noted that reinforcement learning is not mentioned here because it is outside the scope of this research and falls within the realm of predictive control of HVAC systems.

**Figure 7:**
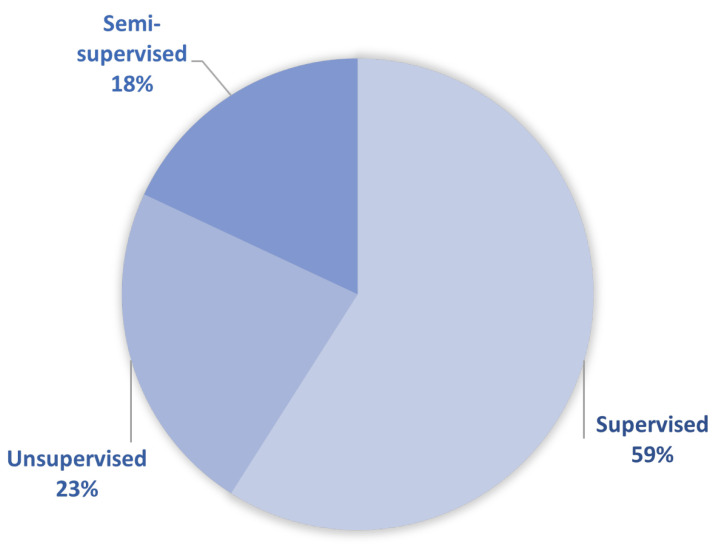
Type of learning used for developing HVAC FDD models within data-driven and hybrid approach.

Figure 7 shows an overview of the mentioned learning types among the selected papers that tackle both the data-driven and hybrid approach. It can be seen that supervised learning is by far the most commonly used by researchers and developers, although annotated datasets are not easy to obtain.

#### 3.3.1. Supervised Learning

Supervised learning only works with labeled datasets, which means that both the input and output data must be known in order for a supervised model to develop a mathematical function that describes the relationship between input and output. Based on this function, the model can predict the output value using previously unobserved input values. Supervised learning can be further divided into classification—for discrete data values, and regression—for continuous data values. This type of learning is often highly interpretable and thus provides a sense of reliability [79]. The papers from the selection that use supervised learning as part of FDD implementation are systematized and presented in Table 4.

Some of the most used supervised modeling techniques in the HVAC field are Multi-Layer Perceptrons (MLPs) [51,56,61,83] and their specific deep learning (DL) derivatives, such as Convolutional Neural Networks (CNNs) [47,60,72,82,84] and Recurrent Neural Networks (RNNs) [57]. There are reports of combining MLPs with other ML models, such as regression trees [61]. Aguilar et al. [56] developed an autonomous cycle of data analysis (ACODAT) that uses Random Forests (RF) and linear regression for a binary classification task to detect deviations in the start-up process, followed by an Multi-Layer Perceptron (MLP) and RF model for behavior prediction. Support Vector Machine-based Multiscale Principal Component Analysis (SVM-MSPCA) was addressed in [91], where the MPSCA technique was used for feature extraction purposes and SVM was applied for fault diagnosis. In addition, various Bayes-based algorithms have been mentioned as successfully applied in the selected literature: Bayesian classifier [90,92], diagnostic Bayesian network [86,94], Bayesian inference with Markov Chain Monte Carlo [85], and Naive Bayes [90] with combination of decision trees (DTs) and RF [55]. Other popular models were: DTs elaborated in [88,95], RF [68,87], a hybrid RF with SVM [59], classifier chains integrated with RF [58], and multi-class SVM [77].

Algorithms that are in the minority but are nevertheless used in the context of supervised learning for HVAC FDD systems are: extreme gradient boosting (XGBoost) [46,93], supervised auto-encoder (SAE) [53], diagnostic multi-query graph (DMG) [75], hidden Markov models (HMM) [74], and ensemble models such the ones based on k-nearest neighbor (KNN) [64] or based on boosted regression trees (BRT) [80].

#### 3.3.2. Unsupervised Learning

Unsupervised learning is used to find patterns in data when the data are unlabeled. Unsupervised modeling does not attempt to predict output as does supervised learning, but instead seeks to find relationships between data instances based on common patterns. Further categorization of unsupervised learning techniques includes clustering and association. By implementing clustering, we obtain grouped data instances that share similar patterns. Association, on the other hand, provides information about the underlying rules that make up the structure of the data.

Selected literature filtered by unsupervised learning type is shown in Table 5. Clustering methods such as k-means with squared Euclidean distances, Ward’s linkage with Euclidean distances, and Gaussian mixture model (GMM) clustering have been utilized in [45,65,70]. Next, association rule mining (ARM) methods were utilized with FP-growth algorithm [96,97], episode-based association methods [69], Apriori, ECLAT, FP-growth [95] and TARM utilized with cSpade algorithm [98].

Other more specific algorithms found in this context are the following: a feature extraction model called three-way data-based kernel Slow Feature Analysis (SFA) [52], conditional Wasserstein Generative Adversarial Nets with optimized ensemble learning quality control protocol [67], and autoencoders with long short-term memory (LSTM) [42].

#### 3.3.3. Semi-Supervised Learning

Finally, semi-supervised learning refers to problems that do not fit either a purely supervised or an unsupervised model. When unlabeled data are combined with a small amount of labeled data, learning accuracy can be significantly increased compared to unsupervised learning, while time and cost are lower than for supervised learning.

Semi-supervised learning is often used by implementing a Generative Adversarial Network (GAN), which can provide a larger amount of data and classes balance with generating a synthetic dataset [43,44,54,63,102]. The single-class (binary) classifier (SVM) is also a popular method, in which a model is trained only on healthy datasets that have been generated by GAN [44,66].

Semi-supervised learning is not exclusively tied to GANs, but can also be combined with unsupervised algorithms—such as ARM, or with supervised approaches—such as Classification and Regression Trees (CARTs) [98]. Dey, Rana and Dudley [76] implemented a framework with novel feature extraction technique that derives non-redundant and informative values about system properties. This helps the main classifier, the multi-class SVM model, to detect faults in the system.

Selected literature, grouped by type of semi-unsupervised learning, is listed in Table 6.

### 3.4. Hybrid Approach

The limitations of white-box models can be improved by combining the previously described approaches, resulting in a hybrid approach, also referred to as a gray-box model. For example, by developing a physics-based model, such as a simulation model in TRNSYS, it is possible to represent the complex thermodynamics of a building system. However, it can be very challenging to collect all the relevant information about a building’s properties and accurately model its dynamics. In this case, the model can become overly complex and it is therefore advisable to combine it with another approach – for example, a data-driven one. In this case, the information about the building’s characteristics does not need to be extensive, but it does need to be supplemented with sensory data measurements [15]. Measurements can improve resilience by reducing noise, uncertainty, and modeling requirements, which can lead to better predictive performance that in some cases outperforms data-driven techniques [39].

According to the work reviewed, it is common to use physics-based models to generate synthetic data (both faulty and healthy data), followed by data-driven modeling, i.e., supervised learning of fault prediction models [80,81,87,88,91,92,93,102]. Simple physical models were used by Chintala et al. [104] where the Kalman filter in EnergyPlus was tested using only thermostat and outdoor temperature to perform FDD of equipment deterioration. Dowling and Zhang [90] investigated the transferability of the model by developing a classifier trained on healthy operational data and estimating a state transition matrix developed using a labeled dataset of HVAC operational and weather data. They tested a Bayesian classifier and a neural network, with the neural network ultimately yielding better results. However, the proposed solution lacks interpretability, which does not inspire confidence in using such a technique for the transferability problem.

On the other hand, the literature suggests that the knowledge discovery approach can also be combined with data-driven approaches, especially pattern recognition techniques [36,96,97,98]. Such solutions employ techniques such as fuzzy logic [99], rule data mining [95,96,97], and clustering [95]. Bayes-based techniques are commonly used as well, e.g., DBN [86,94], Bayesian Inference [85], and Bayesian Method [89]. Finally, there is research on using visualization techniques in combination with pattern recognition techniques, such as structural similarity index measure (SSIM), to find anomalous events in the daily work of HVAC systems [100]. The proposed method consists of a daily RadViz visualization step accompanied by a structural similarity index. The SSIM compares visualizations whose data points are ordered by HVAC components, facilitating the detection of errors in the system.

The use of virtual/soft sensors [80,81,92] and residual analysis [87,88,93,101,103,105] are also techniques worth mentioning in the context of the hybrid approach. All examples from the selected literature dealing with the hybrid approach are listed in Table 7.

## 4. Results Part II: State-of-the-Art Techniques Used in HVAC FDD Methods

In this Section, we give a set of step-by-step guidelines for building an anomaly and fault detection model for HVAC systems. To begin, we discuss the numerous challenges that a researcher should be introduced to in order to better guide themselves in choosing the most viable approach. Next, we provide insight into data preprocessing and feature selection, which are a crucial step in learning predictive models from data. Finally, we review the most commonly used model learning and representation types and describe how to select the most suitable ones depending on the dataset at hand. In addition, workflows for selecting the most viable FDD approach and model type are illustrated to provide a concise overview of the text.

### 4.1. Challenges Tied to Choosing an FDD Approach

In attempting to design a model, a researcher must first be familiarized with the challenges tied to a building’s HVAC system automation in order to identify the crucial views for solving a problem, whether it is related to energy savings, optimizing occupant comfort, early detection of system component degradation, or other.

One of the main obstacles in making additional advances in these fields is the scarcity of labeled datasets, which was already mentioned in Section 3.3. Data-related problems can also be described as data quality problems caused by insufficient sensor activity, sensor degradation, or limited buildings’ sensor quality, variety and positioning. To improve data quality, it is important to preprocess the data to remove outliers, redundancies (uninformative correlations), and noise.

Next, the quantity of data available is very important as it sets the upper bound on the complexity of the learning models allowed, as well as the need for using feature engineering. Moreover, conclusions drawn from experimental evaluation of developed models are stronger if more data is used. If insufficient quantities of labeled data are available, we can use simulation software or data-driven techniques to generate synthetic datasets.

Another problem that is often overlooked involves using feature selection, which contributes to reducing model noise and is an essential part of modeling a solution. Other problems specifically related to data-driven or hybrid approaches include: lack of interpretability (understanding and trusting the inference process), transferability (reusing the model in a new environment) and adaptability (reflecting the changes in the underlying distribution) of a model.

There are also issues related to the lack of research on estimating the severity of faults in component degradation. It is important to diagnose specific failures to give users insight into when to upgrade and repair a component in the future. Issues such as advanced controls and system maintenance over time are also under-researched topics that require more attention.

Finally, HVAC data are time-series data that are collected on a large scale every day. Therefore, it is critical that the FDD system is fast enough to detect errors in continuous and fast data streams. FDD approaches are expensive to implement because not every building has the proper infrastructure for collecting large amounts of high-quality data (e.g., sensors and a database system for storing historical data), and labeling the data is usually costly and labor-intensive. For this reason, FDD approaches are not commonly used in commercial buildings.

#### 4.1.1. Choosing the Appropriate FDD Approach

**Figure 8:**
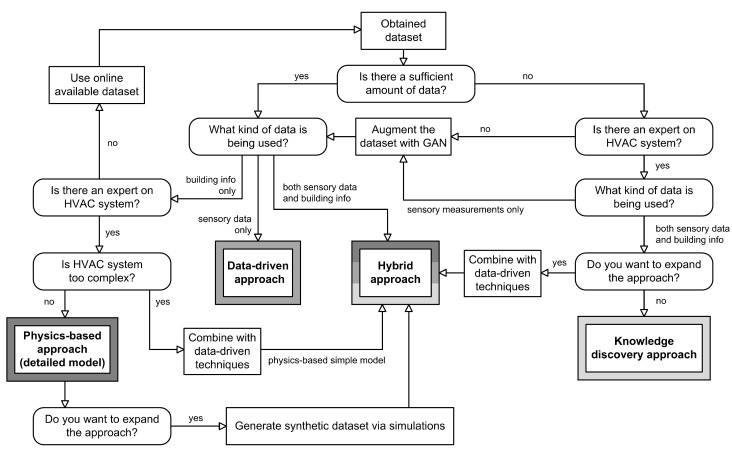
Workflow: Selecting the appropriate FDD approach based on available resources and target goal, taking into account common issues occurring in HVAC systems.

We can use the potential HVAC modelling issues described earlier as a guide to choose the right model, as shown in Figure 8. Key information such as the amount of data and their sufficiency in collected physical details of buildings and/or sensory measurements, the complexity of HVAC and the expertise of the researchers have a great impact on the selection of the approach.

Based on the dataset obtained, it is important to check if the amount of data available is sufficient for the chosen approach. Both data-driven and hybrid approaches are best suited for larger datasets, while the knowledge discovery approach is best suited for datasets smaller in size. Next, we determine what types of data will be used. The data can consist of sensory measurements, physical building information, or a mixture of both types. A combination of both types offers a wider range of possibilities. For example, researchers may choose to work with sensory data only, which suggests a data-driven approach, or they may use a physics-based approach if the sensory data are not of the highest quality. However, they can also choose a hybrid or knowledge-based approach if both types of data are available to use. In addition, the research team may or may not include experts who can detect changes in the behavior of specific HVAC systems and identify, label and draw conclusions regarding anomalies. For physics-based approaches and knowledge discovery approaches, it is advisable to include an expert in the team. The expert can also establish a collection of rules for a particular HVAC system and create an expert rule-based system. However, experts are not required for a model to be successful, as data-driven approaches can work without an expert in the HVAC domain. Moreover, HVAC systems can be more or less complex, which makes a difference when choosing a physics-based model. The simple physics-based model is suitable for complex systems as it can improve efficiency by using optimization methods, while the detailed physics-based model is suitable for simpler systems as it cannot use optimization methods.

After the idea behind the selection of the appropriate approach is established, there are still ways to expand on it. Firstly, if the physics-based approach is the only appropriate approach to choose, it can be extended further by simulating synthetic data that include sensory measurements. This way, researchers can combine data-driven techniques and ultimately switch to a hybrid approach. Furthermore, if the amount of data is insufficient for choosing the data-driven approach, it can be artificially increased using a GAN, which is discussed in more detail in Section 4.1.2. Lastly, the expansion of knowledge-discovery approach can be transformed into hybrid approach with data driven techniques such as Bayesian network, ARM, and other examples mentioned in Section 3.4. Lastly, if none of the suggestions is applicable, there is always the option of including available online datasets, such as ASHRAE, which offer labeled datasets that are often used in a variety of research papers.

#### 4.1.2. Generative Adversarial Networks

When using HVAC data for developing an FDD system, we should note that faults are normally occurring very rarely. Therefore, a typical fairly large raw HVAC dataset contains only a handful of records corresponding to system malfunctions. This makes the problem of learning to detect or classify faults directly from data somewhat demanding, because of the distribution imbalance. Namely, during model training the model is fitted to represent the underlying distribution. If a part of this distribution is severely underrepresented, it is likely that the learning algorithm will treat it as noise, thus making the model oblivious to the HVAC faults. To compensate, GANs can be used to solve the problem of data imbalance by synthetically generating data. GANs are mostly known for their use in computer vision, but their implementation spans other domains as well. In HVAC, GANs are commonly used with data-driven methods because they can correct data balance issues, which means generating a balanced dataset if there is not enough faulty or healthy labeled data. Furthermore, if a dataset of the required size is not available, GANs can simply generate a larger volume of data [107].

In FDD applications, there are examples of modified GAN, Wasserstein Generative Adversarial Network (WGAN) and Conditional Wasserstein Generative Adversarial Network (CWGAN). The main reason for these adaptations is that conventional GANs are able to generate instances that belong to only one class, which is an inefficient way to solve the problem of multi-class labelling in HVAC. By adding a condition to GANs, we can generate different types of instances simultaneously without wasting time on training multiple models [108]. Because the convergence process between the generator and the discriminator can be slow, it can be improved by implementing WGAN, which uses Wasserstein distance to increase the speed of convergence [109].

The authors in [54,63,67,73] used GANs with datasets available online: ASHRAE 1312-RP or ASHRAE 1043-RP. In practice, most authors combined GANs with a supervised classifier such as SVM or an ensemble of learners. For example, CWGAN consists of two MLP networks with a stochastic gradient descent algorithm and a rectified linear hidden activation function, as presented by Yan et al. [63]. The framework consists of a rebalanced training pool through a GAN, a model trained on a binary classifier SVM for detection, and a multi-classifier SVM for diagnosis. Moreover, authors performed a CWGAN combined with an ensemble learning quality control protocol [67]. Next, WGAN was used in combination with ensembles and provided the best performance with SVM in [73]. On the other hand, Li et al. [54] used a modified GAN with a self-training schema as the primary classifier for fault detection. Compared to other standard supervised classifiers, the self-training SVM and the self-training MLP, their proposed model achieved the best results.

In summary, we recommend future researchers to consider GANs when they lack labeled data. We would also like to highlight the CWGAN type as it is widely used in this area of study due to its impact on improving model performance.

### 4.2. Dataset Preprocessing

To get the most out of the available data, data preprocessing is often a necessary step in building an FDD system. Data preprocessing can consist of cleaning, integrating, transforming, and reducing the dimensionality of the data [9]. To begin, cleaning the data is the first step and is referred to in Section 4.2.1, where outlier removal is explained in detail. Furthermore, the most commonly used technique for reducing the dimensionality of data is described in Section 4.2.2.

#### 4.2.1. Outlier (Noise) Removal

Outliers are abnormal data points with values that deviate from the rest of the dataset, increasing the variability of the dataset. They can affect the actual results and cause the ML model to miss important insights. Although outliers can also be informative and important to a model’s knowledge [110], we describe them only as errors or noise in the dataset. It is advisable to use specific outlier removal techniques to automatize the preprocessing step, such as: kernel density estimation-based statistical approach (KDE) [96,97], isolation forest algorithm (IF) [62], the rule of interquartile range [95] and Hampel filter method [61,98]. Other published works do not explicitly specify the outlier technique used, most likely relying on manually removing suspicious records where needed [58].

#### 4.2.2. Principal Component Analysis

Data dimensionality is an important characteristic to consider when dealing with HVAC data. In some cases, if the data are high dimensional in the feature space, a problem may arise in which a subset of data instances lacks a meaningful representation of the dataset as a whole. Therefore, it is beneficial to limit the size of the input feature set using dimensionality reduction methods such as principal component analysis (PCA). Such dimensionality reduction methods are classified as feature extraction techniques, which are a subset of the feature engineering process. The main goal of feature extraction is to improve data by transforming it into the most relevant and distinctive form possible, while preserving as much of the original information. This greatly improves the performance and accuracy of models [6]. It is important to note that PCA is referred to as a preprocessing step, not a feature selection step, as it transforms the original dataset into a new, condensed representation. PCA linearly transforms the input space into a compressed representation using eigenvalue decomposition or singular value decomposition (SVD) [79]. In essence, PCA lowers the dimensionality of the input while retaining most of the variation in the underlying distribution. It is the most commonly used modern technique in data preprocessing, having the goal of creating a smaller projection of the dataset before training a model. That way, the trained model normally has lower variance, while its generalization accuracy is increased.

PCA has mostly been used as a feature extraction or dimensionality reduction technique [66,102]. For example, in [66], the data were reduced to a two-dimensional space by PCA and then used to train a one-class SVM for fault detection. Similarly, in [102], important features were retrieved for training an SVM using a multiscale interval PCA extension, which is a more reliable approach for extracting the most important features. Next, a study was conducted where the implementation of multiscale PCA improved the classification of an SVM model [91]. On the other hand, Yang et al. [55] investigated the impact of PCA on a predictive model and it was found that the use of PCA significantly lowered the predictive accuracy of the model. Furthermore, PCA was used as a method to detect faults in [44]. The proposed technique uses kernel principal component analysis (KPCA) to capture the normal operating conditions of the system. The learning approach is successful in dealing with nonlinear events due to the use of the Gaussian kernel, which guarantees high accuracy attributes through a self-optimization mechanism while maintaining sufficient generalization. Next, in [48,101], the authors combined PCA with Hotelling’s T2 and square prediction error statistics (SPE). Together with these two statistical methods, unfold-PCA is used as a batch projection method for alarm generation [48]. In another research paper, Hassanpour et al. [101] used PCA to detect errors. Several hybrid PCA models were developed using residuals and temperature measurements. The hybrid PCA model is used to calculate the above statistics to monitor the process and detect deviations from expected behavior.

Even though PCA has mostly been used as the main dimensionality reduction technique in HVAC, PCA can still be outperformed by nonlinear algorithms. The main limitation here is that PCA is a linear algorithm that brings unusual data points far apart into a lower dimensional form when transforming nonlinear data, but does not retain the intrinsic shape of nonlinear samples. For this reason, these techniques do not model the fundamental nonlinear relationships. Therefore, it is important to note that PCA is a linear algorithm that, along with HVAC’s non-linear data measurements can be outperformed by non-linear algorithms such as Isometric Feature Mapping (Isomap), Kernel PCA (KPCA), Local Linear Embedding (LLE), t-distributed stochastic neighbour embedding (t-SNE), Autocoder and Laplacian Eigenmaps. The official comparison of mentioned nonlinear algorithms with PCA was made in [111], where the authors concluded that KPCA and t-SNE outperformed the other techniques, but the performance strongly depended on the different types of datasets and the tuning of the hyperparameters.

### 4.3. Feature Selection

Feature selection is a crucial step towards eliminating correlated data, thus reducing model complexity, which often helps improve its generalization properties and prevents overfitting. Essentially, it is a part of feature engineering where the main task is to identify the minimum subset of features that have the greatest impact on improving model performance. However, when implementing a feature selection technique, it is important to be aware of the potential loss of information if the selection is not performed correctly. Feature selection can be supervised or unsupervised, where the former uses the target variable to eliminate irrelevant input variables, while the latter relies only on the patterns encoded in the input data. According to [6,112,113,114], feature selection can be divided into three main categories: filter methods, wrapper methods, and intrinsic or embedded methods. Those methods are most commonly used in a supervised setup, however, unsupervised examples exist as well. Before modeling, filter methods select features from the original dataset. The aim is to evaluate the variables and their sensitivity to error using statistical measures to determine the correlation between the input variables. These methods are less prone to overfitting and work much faster than the other methods discussed. Furthermore, the choice of a filter method depends on what kind of data the inputs and outputs are. In the field of HVAC, we can expect both (continuous) numerical as well as discretized data (either ordinal or nominal) as inputs and outputs. For regression problems it is best to utilize a correlation coefficient for linear correlation, such as Pearson’s algorithm, or rank-based techniques for non-linear correlation. Furthermore, numerical inputs and category outputs are an example of a classification problem in which typical techniques are correlation-based, however in this problem, the categorical target must be considered. ANOVA Correlation coefficient and Kendall’s rank coefficient are the two most common examples. Some notable utilization of filter methods in the literature can be seen as filtering by non-local means algorithm [84], Pearson correlation coefficient [62,106], Relief algorithm [59], and steady-state method based on the change rate of the reference variable [53].

Next, wrapper methods use the model in the selection process and attempt to analyze all features to determine the most discriminative subset in terms of performance from the entire dataset. The feature selection procedure is evaluated on the ML algorithm that we are attempting to use to model a particular dataset. Because they can be computationally demanding they are far from ideal for big datasets, but they often provide the best subset of features, compared to filter methods. There are three types of wrapper methods: (1) forward selection, where we add a feature in each iteration until an additional new variable no longer improves the performance of a model, (2) backward elimination, where we input all features and eliminate them in each iteration, and finally (3) recursive feature elimination, which is essentially a greedy optimization algorithm [112]. In the literature, these methods are not prevalent since they are time-consuming, but some authors mentioned them in a form of cost-sensitive sequential feature selection (CSSFS) [63,67,73] and cross validated recursive feature elimination [93].

Finally, embedded methods choose features throughout the model-building process. The model evaluates input variables by assigning weight coefficients to each feature and selecting characteristics that lead to greater model accuracy. The time complexity is midway between the filter and wrapper methods. Some examples are regularization techniques such as Lasso and Ridge regression, but it can also include a hybrid of filter and wrapper methods in a parallel or a two-step serial combination. Regularization in particular is often neglected in research papers and in reviews. From the explored available literature there is only a handful of articles that mention the use of regularization techniques incorporated into their models [43,47,49,50,51,53,54,57,64]; however, they fail to present it consistently. Some authors did explain in more detail how they incorporated regularization, such as stating the dropout rate [43,49,54]. Some combinations were made as follows: dropout with batch normalization [53], dropout with early stopping [49], and dropout with Ridge regularization (L2) [57].

Essentially, regularization is a type of optimization in which model parameters tied to less informative features are pushed towards zero during model training. By balancing between the empirical model accuracy (based on training data) and the overall sizes of individual parameters, regularization reduces the chance of overfitting the model to training data because it prevents learning unnecessarily complex models. For example, let us assume a non-regularized model is not able to generalize well to new data. Regularization significantly reduces model variance without noticeably changing its bias. Thus, the tuning parameter, regularization rate, controls the effects on variance and bias. As its value increases, model parameter values decrease on average, thus lowering the model’s variance. This increase is beneficial up to a point, as it only decreases the variance and prevents overfitting without giving up important data properties. However, after this point the model is more difficult to adapt to healthy informative data, leading to increased bias and underfitting. Therefore, the value of the parameter must be carefully chosen. The most popular regularization methods are Lasso and Ridge regression. In both methods, the coefficients are calculated by finding the first point where the elliptical contours intersect the range of constraints. Since lasso regression creates a diamond shape in the graph for the constrained region, at least one of the coefficients becomes 0 when the elliptical regions intersect with these corners. This is not possible with ridge regression because it has a circular shape, so the values can be reduced to zero, but never become zero [79].

On the other hand, unsupervised feature selection can be implemented using filter methods that are mainly used by clustering algorithms [61,95]. Clustering techniques are presented in more detail in Section 4.4.6.

In general, the main goal of feature selection is to use careful systematic testing to identify what works best under specific circumstances. It is advisable to always try out different models based on a variety of data selected by various statistical metrics to see what works best for a specific problem.

### 4.4. FDD Model

In this Section, we present the most common, yet effective, modeling techniques used to implement FDD models using the data-driven approach. We explain their characteristics using research results from the reviewed literature. Finally, we compare their advantages and disadvantages and provide an intuitive flowchart for selecting the appropriate model for an FDD problem.

#### 4.4.1. Support Vector Machine

Support vector machines (SVMs) [79] are one of the most commonly used modeling approaches for FDD in HVAC. Essentially, an SVM is a binary maximum margin classifier, defined by a boundary that is unit-separated from the nearest instances of both classes using the simplest possible representation (regularization effect). The dimensionality of the boundary depends on the number of data features. The goal of the model is to determine the boundary ensuring the largest distance between it and the closest data instances of the two classes. Increasing the boundary distance provides some reinforcement and allows for a more accurate categorization of the data instances. To successfully model an n-dimensional space, we pinpoint those data instances that are closest to the boundary (i.e., support vectors) and influence the boundary’s position and direction. Because SVMs are linear classifiers, more complex distributions can be encoded seamlessly using kernel functions. A kernel function is basically a computation of high-dimensional relations of input data without the need to explicitly transform the data. It reduces the computational cost by avoiding the transformation of the data, and allows the computation of relations in an unbounded number of dimensions. Polynomial kernels and radial basis function kernels are the most commonly used [79]. For regression problems, the alternative to SVM is called Support Vector Regression (SVR). Here, support vectors are utilized to form the linear hypothesis [115]. Because SVM is inherently a binary classifier, multi-class SVM (MCSVM) can be used to classify multiple classes. MCSVMs are typically implemented by incorporating several binary SVMs. There are numerous methods for solving multi-class classification problems for SVM, including Directed Acyclic Graph (DAG), Binary Tree (BT), One-Against-One (OAO), and One-Against-All (OAA) classifiers [116]. A semi-supervised type of SVMs is also mentioned in the literature, called self-training SVM. Essentially, self-training algorithms work by iteratively learning a classifier by assigning pseudo-labels to a set of unlabeled training instances with a margin greater than a certain threshold. This method is resistant to class imbalances and is able to efficiently use both labeled and unlabeled data, even when the class distribution is highly skewed [117].

An SVM was used as a regression model in a hybrid approach where its inputs were provided by the physics-based model. The SVM performed poorly when compared to alternative regression models such as Gaussian process regression (GPR) [81]. An SVM and a self-training SVM performed worse than a GAN, but also showed their advantages over other supervised classifiers in [54]. Tun et al. [59] used an SVM as an additional classifier that improved the accuracy of an RF model. In their workflow, RF was better suited for working with large amounts of noisy data and extracting features. The extracted features were then passed to the binary SVM classifier. SVM was better suited for processing low-dimensional data, and in this workflow SVM improved the accuracy of classification. In addition, an SVM was implemented as both a binary classifier and a multi-class classifier. The binary classifier was used to detect if an instance represents a fault first. If the detection was positive, the instance was further classified using the multi-class classifier to diagnose the fault next [63]. Furthermore, Yan et al. [67] tried an ensemble of learners and found that SVM-RF-MLP had the best performance among the other ensembles. Martinez-Viol et al. [66] presented a binary SVM model that demonstrated its resistance to outliers during training. When the number of features and training instances is small, an SVM is considered a very strong technique that can achieve good accuracy without requiring manual fine-tuning of the model. SVM can be combined with a multiscale interval PCA extension that leads to more accurate classification [91]. Multi-Class Support Vector Machine (MC-SVM) was used to create an automatic FDD system based on categorical data to make the building smarter [77]. The same authors have previously conducted research on the implementation of MC-SVM where they investigated linear kernel SVM (i.e., not transforming the input space), quadratic kernel SVM and three variations of KNN models. Linear kernel SVM proved to be the best-fit model among the five tested methods [76].

Although they can work as multiclass classifiers, SVMs work best on low-dimensional data and are preferably used as binary classifiers. They are efficient and, when combined with other models, can increase modeling accuracy.

#### 4.4.2. Artificial Neural Networks

ANNs are models that mimic the processes of the brain to express correlations in highly nonlinear data distributions. Similar to the brain, an ANN consists of neurons (or nodes) that encode outputs as functions of the inputs. Its structure consists of an input layer, one or more processing layers (hidden layers) of connected nodes and an output layer. Model weighting is adjusted until the model has the smallest possible margin of error. Due to their structure, ANNs can encode more complex representations by adding more hidden layers, which consequently enables them to learn useful feature embeddings (i.e., perform DL).

In general, DL is a subset of ML, enveloping those ML techniques that rely on learning latent representations in an end-to-end optimization process—as opposed to non-DL methods that usually rely on feature extraction to simplify the modeling distribution. Essentially, a DL model consists of a collection of basic building blocks of particular types, some of which can be adapted by learning their parameters (i.e., weights) from data to improve overall model prediction accuracy. When a composition is called “deep”, it means that numerous building blocks of that type are stacked in a hierarchy of increasing complexity. ANN building blocks are expressive enough to be used for building DL models. Therefore, they are commonly the first choice when one is interested in building a DL model.

In addition to an ordinary fully-connected feed forward MLP, some of the most popular DL alternatives are CNNs and RNNs. CNNs are a DL modeling technique typically used in computer vision to analyze and recognize visual input, such as digital photographs. RNNs are a DL modeling technique that tolerates and benefits from the sequential nature of inputs, just as CNNs accept the spatial structure of image inputs. In summary, they are intended for the analysis of time series data, event histories or temporal sequences [118]. Additionally, a form of self-training MLPs were mentioned in the literature [43,54], analogous to the term explained in Section 4.4.1.

Aguilar et al. [56] created ACODAT, in which an MLP was employed for behavioral prediction which determines the quality of a multi-HVAC system setup. Next, twenty-two different neural network architectures were analyzed, and one was chosen to be coupled with a physical model in order to provide an accurate simulation tool capable of producing faulty and fault-free datasets that can be evaluated and used to determine the impact on energy consumption [83]. Moreover, MLPs and self-learning MLPs performed worse than GANs, but they also outperformed other supervised classifiers [54]. In [43,49], the authors explore the value of a semi-supervised self-training neural network which in the end shows its effectiveness and the possibility of enhancing unseen fault detection rate in AHU operations. Zhu et al. [51] succeeded in creating a transferable model by developing a domain adversarial neural network (DANN). DANN combines domain adaptation with deep feature learning in an adversarial training process, so that final classification judgments are based on features that are both discriminative for the target task and invariant to changes in the domain. Next, Piscitelli et al. [61] combined a MLP and a regression tree built using the algorithm CART (CART, MLP) with an aim to detect anomalous patterns and trends in energy consumption. The combination ensured the interpretability of MLPs. CNNs aim in [82] is to analyze the correlation between the system variables and to take into account the temporal influence of time series signals without risking the recognition time. The proposed approach eliminates the need for advanced data preprocessing and is computationally efficient. Furthermore, to overcome the disadvantage of DL black-box model interpretability, Li et al. [60] proposed a novel explainable DL-based fault diagnosis method suitable for HVAC systems. CNNs have been improved to be multi-scale and provide better feature extraction capabilities for FDD of RLT devices [47]. In [84], the proposed framework combines the rule-based method and the CNNs-based method. Faults can be identified with a high accuracy of 99.15%, including fast online detection within 2 min. Miyata et al. [72] used system data visualizations directly as model inputs for the CNN-based FDD approach. For fault diagnosis, a novel FETCN technique was presented. Initially, characteristics describing the dynamics and changes in the chiller system are retrieved and improved using the statistical pooling approach. Following that, a Temporal Convolutional Network (TCN) classifier is used to analyze the features and diagnose the problem [50]. In [57], Stacked deep RNN was the ultimate model for fault diagnosis of HVAC systems over a long period of time. Additionally, a variant of the artificial recurrent neural network called LSTM was proposed and it performed the best in prediction performance in comparison to the XGBoost method when the time-series data fluctuated greatly. Next, a framework was developed by Zhu et al. [103] that provides guidelines for implementing predictive maintenance of building installations. When the data are collected, LSTM network is used to predict faults. Yun et al. [53] wanted to see if a neural-network-based FDD model could yield substantial inferences for input variables using SAE. The real-time monitoring data are used as model input. The offline model is essentially trained to predict fault-free or faulty class, and additionally generates a reliability value for every predicted label, which is then analyzed using threshold settings.

To summarize, compared to other models, neural networks have seen the highest exploration and utilization in recent years. It is clear that MLPs, CNNs, and RNNs may be successfully applied within FDD solutions. However, for predicting time-series data, RNNs, notably LSTMs, are preferable. CNNs, on the other hand, are said to have great accuracy and exceptional capability in learning complicated functions and interdependence from any given input. Keeping this in mind, CNNs require a large quantity of data to be efficient and accurate when compared to other networks. Finally, MLPs are the most basic sort of network that may be combined with other models such as CART or SVM.

#### 4.4.3. Decision Tree

DT models can be used both for classification and regression tasks. DTs consist of root nodes, decision nodes and leaf nodes. A root node, also called a parent node, represents the entire population and divides the data into two or more nodes. When developing a tree, decisions must be made about which features to include as input, the conditions for splitting and when to stop further branching of the tree. In addition, trees grow randomly. Therefore, pruning techniques are used to improve the performance of the tree by eliminating branches that use less important features. By reducing model complexity up to a point, we reduce overfitting and increase its generalization accuracy. Entropy and information gain are important for branch partitioning. Entropy measures the degree of unpredictability in a series of events and serves as an estimator. These estimates are then calculated using the information gain formula, which provides a sense of certainty about the class of a target variable. A DT model is usually easy to develop and has high interpretability as it can be easily visualized and explained. Moreover, the selection of features is implicit. The main problem is overfitting, as a tree can become an overly complex model that lacks generalization to new data. On the other hand, severe bias can occur if the imbalance of classes in the dataset is not taken into account [119].

DT has been used as a reference model to interpret MLPs in [61]. Furthermore, authors examined DTs using a combination of temporal ARM in [98]. DTs were used for detecting faults on the non-transient period of the dataset. Their rule-based approach provides interpretability and the data-driven side of the methodology enables automatic learning of operational patterns. However, the granularity of the ASHRAE 1312-RP dataset used had to be reduced to obtain optimal model performance. In addition, the DT was used to classify data categories at the zone level, where they were combined with association methods [69]. In system health monitoring, due to its simplicity, DT model was implemented to distinguish normal operation from abnormal [55]. The same classification of normal and abnormal data was performed in [95] as a post-mining tool. On the other hand, the generated residuals were classified using DTs in [88]. The applications of CART models are mentioned in several articles [61,98]. Furthermore, many ML methods were tested on synthetically created as well as real-world datasets where boosted trees produced the best results [80]. For outlier detection and fault diagnosis, a hybrid deep forest technique was proposed. For outlier detection, the IF approach is used with Pearson’s correlation coefficient. Additionally, a DL model—cascade forest (CF) based on DTs is suggested for fault detection of HVAC systems, achieving high precision accuracy in low-dimensional data [62]. This model does not require a complex hyperparameter optimization strategy and provides the highest accuracy when compared to Back Propagation Neural Network (BPNN), MLP, SVM and LSTM.

DTs used for classification tasks are more commonly used than regression trees because the main objective is to detect errors. On the other hand, regression trees are better suited for model predictive control (MPC), where the goal is to predict certain values such as time to failure or energy consumption. In summary, the classification DT model performs best with a smaller number of classes and has the advantage over other models for its simplicity and interpretability. Furthermore, outliers do not have a significant impact on the model and the same variables can be used multiple times in different parts of the tree, allowing a model to reveal dependencies between groups of variables.

#### 4.4.4. Random Forests

If a DT model exhibits too much variance in spite of using appropriate regularization techniques, it can be replaced by implementing an ensemble of DTs called the RF model instead. An RF model predicts a class by averaging the results of multiple trees, and its accuracy improves as the number of trees increases. This is accomplished through a process of sampling with replacement called bagging coupled with random feature selection at each tree-building step to train ensembles of trees for attaining higher predictive accuracy. Essentially, it is a model that can handle encoding more complex distributions by using highly expressive individual models whose variance is in turn constrained through voting during inference. However, training an RF model requires more computational time compared to a DT. Moreover, they do not handle sparse data well and cannot extrapolate properly, so classification random trees are used more often than regression trees [79].

RF is studied and compared with an autoregressive (ARX) model to predict fault-free operation by predicting the total heating capacity of a building, followed by the detection of faults using residual analysis [68]. RF is more difficult to interpret, but has minimal problems with overfitting and provides an efficient nonlinear modeling strategy. Parzinger et al. [87] extended their earlier research by developing an algorithm to determine the best decision rule for determining errors. Wu et al. [58] employed a novel hybrid method of classifier chains with integrated RF method (CC-RF) to treat concurrent faults in RLT units as a multi-label problem. Another novel hybrid method was proposed in [59], wherein a combination of RF and SVM (RF-SVM) classifiers was utilized. RF is used to extract the most important features, which improves the generalization ability of the proposed FDD system.

RF is often used in hybrid approaches to improve accuracy. It is advisable to use this approach when a large number of classes needs to be predicted. Although lack of interpretability can be a problem in the field of HVAC system monitoring, it has shown good predictive performance.

#### 4.4.5. Extreme Gradient Boosting

Some shortcomings of RF modeling can be solved using Extreme gradient boosting (XGboost)—an ensemble learning technique based on gradient boosting, a potent algorithm in which each consequtive predictor corrects the cumulative error of its predecessors. In XGBoost, particular models that form the ensemble (DTs) are built sequentially, on the residual of the previous models. These individual classifiers/predictors then ensemble to form a strong and more accurate model [120].

In [46], a hybrid reference model called multi-region XGBoost is used as a classifier, which integrates a type of DT called CART. Results of this model show its high accuracy in identifying errors, indicating that it generalizes well and is a reliable and efficient model for FDD purposes. The model outperformed the SVM and the regular XGBoost model. Furthermore, XGBoost was employed as a prediction model for energy usage in [93], where it was combined with a novel dynamic threshold approach for FDD. The method detects fault occurrences and dynamically modifies the threshold value based on the real-time moving average and moving standard deviation of the forecasts.

In spite of not being used commonly for HVAC FDD, XGBoost models are efficient with large datasets, can handle class imbalances and can outperform SVMs, among others, in FDD tasks. Moreover, due to its foundation in the decision tree boosting principle, which employs a sampling technique, XGBoost models are insensitive to distribution skewness.

#### 4.4.6. Clustering

Even though we focused mostly on common supervised types of learning methods, for unsupervised type we singled out clustering as a proposed method if the dataset is unlabeled. Clustering involves the automatic identification of natural groups of data instances. A cluster can be defined as a dense region in the feature space where observations in one area are closer to one group than another. Clustering can be used as knowledge or pattern recognition due to its ability to discover relationships between unlabeled data instances. In addition, it can also be used as a feature engineering method where groups of clusters can be labeled as one cluster. An expert may be required to evaluate the identified clusters, as the results may be subjective to a particular research area. Clustering algorithms attempt to find dense regions of observations using similarity or distance metrics between instances in the feature space. Therefore, data must be scaled before applying clustering techniques. It should be noted that some clustering methods require a parameter for the number of clusters the user wants to find. Other algorithms require a parameter that defines the minimum distance between data instances in order to appropriately group the data [119].

In the literature, a multi-regional XGBoost model (based on CART) was developed using a mean-shift clustering method [46]. Clustering based on the “Follow The Leader” algorithm was performed, followed by frequency analysis to eventually label anomalous data [61,98]. A study in [70] proposed a ML-based multi-stage automatic fault detection system focusing on FCU subsystem analysis. The method uses sequential two-stage clustering to identify abnormal behavior. The cluster analysis based method was performed in [65], where the faults were detected by Ward’s linkage method with Euclidean distances in the data, which were otherwise missed by the operational staff and previous commercial FDD tools. Next, anomaly detection at the zone level was performed by using various clustering methods, such as k-means, Gaussian mixture, and agglomerative clustering algorithms. The best algorithm was selected based on the Calinski–Harabasz index [45]. Feature selection was performed by clustering analysis that included agglomerative hierarchical, k-means, and PAM algorithms [95]. In a hybrid approach, a physical model was built and evaluated using the clustering technique by measuring the distances between instances to distinguish between normal operation and four types of faults [26].

From the literature reviewed, it appears that clustering can be used in fault detection, data labeling, feature selection, and as an evaluation technique. In particular, Multi-Objective Clustering-Rapid Centroid Estimation (MOC-RCE) has been shown to be the most efficient feature selection method. When dealing with unlabeled datasets, clusters are a viable option that should be explored and experimented with.

#### 4.4.7. Association Rule Mining

ARM is a data mining technique for finding common patterns, correlations, relationships or causal structures in datasets containing non-numeric, categorical data. The goal of ARM is to find rules that allow predicting the occurrence of a particular element based on the occurrence of other elements in the events. An association rule consists of two components: an antecedent (if) and a consequent (then). An antecedent is something that occurs in the data and a consequent is something that is associated with the antecedent [119].

In the reviewed literature there have been several implementations of this method [69,95,96,97,98]. For example, the work described in [98] implemented a two rule-extraction method including DT and TARM. TARM methods were used for detecting faults in transient periods. On the other hand, the work described in [69] focused on metadata inference without any semantic information. The authors developed a zone level inference method which included classification by DT and an association method—episode-based association. After the data points are classified, the association method discovers the functional relationships among these point classes by grouping the data using different matching strategies. Next, authors in [95] proposed an ARM anomaly detection and dynamic energy performance evaluation method for an HVAC system to evaluate multiple energy performance metrics of individual buildings in a short time interval (i.e., every hour). They used clustering techniques where the ARM method was applied to each data cluster. The post-mining process was further evaluated using DTs. Furthermore, to automatize the selection of rules and improve the performance of the ARM method, authors in [97] improved the post-mining of associated rules by developing a rule of comparison-based post-mining. It consists of association rule grouping, association rule normalization, association rule comparison and expert rule analysis. Similarly, post-mining method was improved in [96] by proposing the implementation of a fuzzy analytic hierarchy process. The post-mining method consisted of three criteria and six sub-criteria to evaluate the value of each association rule. Fuzzy set theory was used to evaluate the sub-criteria of association rules. Finally, the analytical hierarchy process was applied to determine the weight of each criterion and sub-criterion, resulting in an overall evaluation of the rules. Finally, k-means clustering algorithm was used to classify the rules based on their Euclidean distance.

To summarize, ARM is often combined with clustering, or even supervised methods such as DT (where applicable). ARM is capable of extracting association rules between variables from massive operational datasets. Even though ARM can be efficient in creating rules, the main drawbacks of ARM include, among others, the excessive generation of non-informative rules that are difficult to filter out if post-processing is not automated. Furthermore, the majority of ARM algorithms (Apriori, Eclat and FP-growth) work with categorical data only. Seeing that HVAC datasets consist primarily of numerical measurements, they must be converted into categorical data which requires additional data preprocessing and tuning [97].

#### 4.4.8. Choosing the Right Model

**Figure 9:**
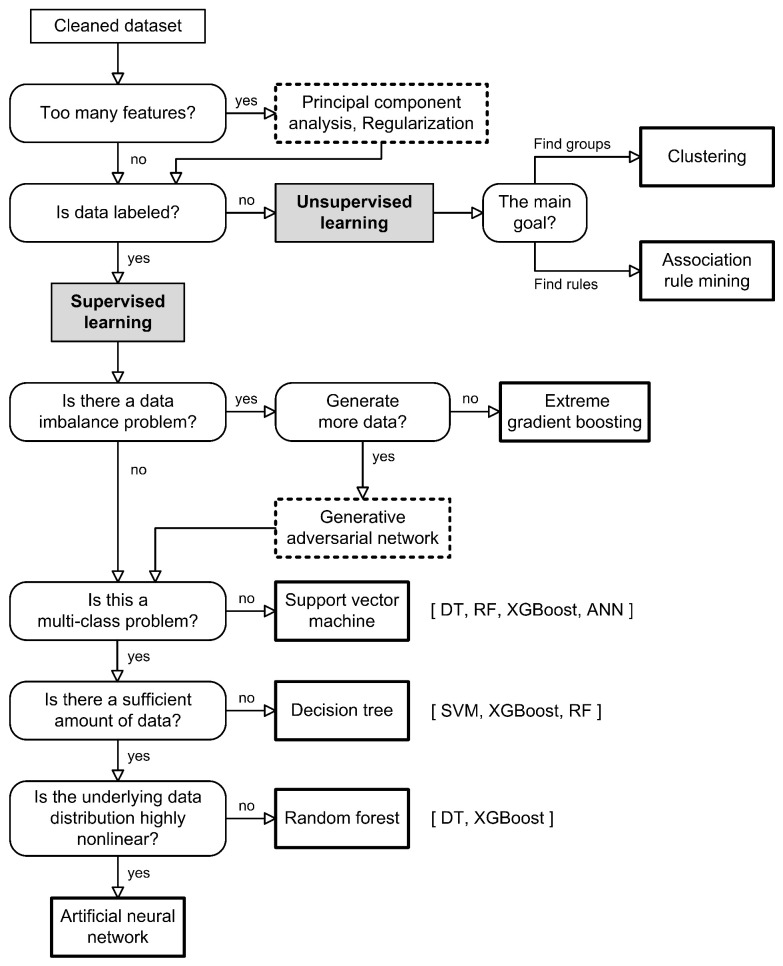
A diagram depicting the process of developing an FDD system, to help with choosing appropriate modeling techniques. Alternative techniques to those recommended are presented in square brackets.

Figure 9 shows the proposed workflow for developing a model for HVAC FDD.

First of all, we assume that we have at our disposal the data from the sensor infrastructure in a building (e.g., a hotel). The usual data that a typical monitored HVAC system can provide in such a context are: user-set desired temperature, current achieved temperature in the room, HVAC fan speed, HVAC valve status, room occupancy, window status (open/closed), etc. Meteorological data related to the building’s microsite can often be added to this data, whether it comes from an internally installed weather station or is supplied by a third party (vendor). Typical meteorological data that affect the thermodynamics of the building and can be collected include radiation (solar radiation falling on a surface), humidity (the amount of vapor present in the atmosphere), and outdoor temperature. Generally, all of the above data are collected in a time series format that matches the sampling frequency of the sensor readings in the HVAC system.

Next, the clean dataset should be obtained, meaning that the original raw dataset was previously preprocessed, as mentioned in Section 4.2. The preprocessing steps depend on the raw data and may consist of cleaning up outliers and missing values, either manually or using specific methods mentioned in Section 4.2.1. In addition, normalization and standardization can be applied if needed to make data points uniform.

After the target dataset has been cleaned and prepared, it is ready for feature selection. If the number of features is too high compared to the computational power at our disposal, we can use feature selection or dimensionality reduction techniques to obtain a denser version of the same dataset, as a one-time preprocessing step. Alternatively, regularization should be preferred for dealing with noise removal and overfitting. Next, we need to determine whether the dataset is labeled with faulty classes or not. Based on this, we narrow down the choice of algorithms available to us, depending on their learning type:

- Learning from unlabeled data can be performed using unsupervised learning techniques. Proposed methods include clustering and ARM. ARM is normally used when the main goal is to find connections between attributes in the data, whereas clustering is used when the goal is to find relationships within data points.
- For labeled data, if there is an imbalance between classes, we recommend using XGBoost models or GANs. XGBoost models can deal with class imbalance by providing a way to tune the algorithm to pay more attention to minority class misclassification in datasets having a skewed class distribution. On the other hand, GANs can be used to augment the existing dataset by generating a larger amount of labeled and/or unlabeled data. In this way, all upcoming supervised learning techniques will achieve better results because their inputs will be class balanced. At this stage, there are a number of options for creating supervised models:
  – If a binary classifier is needed, SVMs are advisable because they maximize the margin between the neighboring points of opposed classes. All the other models mentioned are also suitable for binary classification, but seeing that SVMs are inherently a binary classifier, it is advisable to try them first.
  – If the number of data instances is not overwhelming, and multi-class classification is needed, DTs are likely the best option since they are highly interpretable, and are therefore reliable to implement in an actual HVAC system. As an alternative, it is advisable to compare DTs with models such as SVM, RF and XGBoost.
  – If the underlying data distribution is highly non-linear in nature, then ANNs offer more flexibility—albeit at the expense of additional complexity—CNN models for spatial data, RNN/LSTM for temporal data, and MLP for everything else. If not, then an RF model may be more suitable for this purpose, because it requires tuning less hyperparameter values.

Although the workflow described above provides a simple way to select appropriate modeling techniques, in practice it may differ slightly depending on the type of the problem researchers are trying to solve. One rule cannot be applied to all FDD problems, so it is important to consider several other things when developing FDD solutions for HVAC systems. This leads us to a parametric and non-parametric categorization of such models. This is a fundamental ML concept for categorizing individual models based on their capabilities. The main difference is that parametric models have a fixed number of parameters, while non-parametric models have an increasing number of parameters related to the growth of the training data. Parametric models such as ANN, clustering, naive Bayes classifiers, and linear and logistic regression (LR) are faster to compute, but can tend to make stronger assumptions about the nature of the data distributions. Non-parametric models such as SVM, XGBoost, DT, CART, ARM and KNN classifier are more flexible, but can be computationally intensive for large datasets. It is also important to note that SVM can only be listed as a parametric method if it is used as a one-class or binary classifier. For highly flexible models (non-parametric models), it is also important to be careful not to overfit by not modeling all minor variations, as these often represent noise rather than important data [121]. This is usually circumvented by the use of regularization.

In order to compare the main features of all the models mentioned, we have summarized their advantages and disadvantages in Table 8.

## 5. Conclusions

This review paper addresses the latest cutting-edge research in the field of FDD HVAC solutions. It is based on a total of 77 representative, high-quality papers that were carefully selected and thoroughly inspected as part of a systematic literature review process.

Approaches to HVAC FDD were presented from a new perspective, proposing a new classification that includes:

- knowledge discovery approach,
- physics-based approach,
- data-driven approach, and
- hybrid approach—which combines techniques from different contexts, with an emphasis on data-driven ones.

Each approach is then explained in detail and analyzed in light of the selected literature in which it is addressed. The main focus is put on the data-driven approach, which includes the models and data preprocessing techniques commonly used in this field. For this approach, both the types of learning algorithms (supervised, unsupervised and semi-supervised) and the special cases of these algorithms used in the literature are presented in more detail. All systematizations and classifications are supported by corresponding tables, which contain the most important information about the reviewed work.

In the second part of the paper, we focused on the state of the art in data-driven FDD methods for HVAC systems. We first explain the underlying problems of HVAC systems, and then propose and argue a workflow for selecting an appropriate FDD approach based on the available data resources and the intended goal. We believe that such a workflow, together with the literature review mentioned above, can help researchers focus more quickly on the right way to solve their problem. This especially holds for researchers and solution developers who are just beginning to explore the field, which in this case can seem quite confusing and very broad at first glance.

Finally, another workflow is proposed to support the development of FDD systems in more detail - starting with the available dataset and ending with the selection of the right modeling methods and techniques. We derived this heuristic guideline based on the large number of investigated solutions and proposals from the literature. It is aimed at all HVAC researchers who want to quickly navigate the world of data-driven techniques. Indeed, we believe that this heuristic can help to make fast yet relevant decisions regarding data preprocessing and modeling FDD solutions.

Some remarks for future research to consider can be summarized as follows:

- Before choosing an approach and a model, the background of the potential problems of HVAC systems must be known. These include: the complexity of the systems, the management and collection of high-quality data about the building and the system itself, and access to high-quality sensory measurements.
- Scarcity of labeled datasets in this area is still an unsolved problem that researchers need to prioritize.
- It is important to develop a model that is transferable and interpretable.
- FDD solutions are under-implemented in commercial buildings because they can be costly and computationally intensive. Therefore, it is important to make the methods more efficient as they use streaming time-series data.
- There is a lack of research regarding errors in detecting the severity of component wear and system maintenance over a period of time.
- Some HVAC subcategories have not yet been adequately researched, such as FCU subsystems.
- Although feature selection is an important step in developing a model, regularization techniques need more attention and implementation in future work.

In conclusion, we hope that this paper will encourage researchers and solution developers to improve FDD methods so that they can be widely used in real HVAC systems. This would open up opportunities for better energy management, take the implementation of smart rooms and smart buildings to a higher level, and ultimately lead to a cleaner environment. In this context, our future work plans consist of developing an FDD solution for FCU subsystems and addressing the challenges mentioned above.

We also provide a Supplementary Spreadsheet (Table S1 ) that contains all the essential information about the related work we used in the SLR process. The corresponding document systematizes the selected papers according to the classification proposed here.

## Figures and Tables

**Table 1:** Brief summary of related work addressing the physics-based approach in HVAC FDD. For clarity, all acronyms are provided in the Abbreviations list.

Target (sub)System	Software	Year	Ref.
Heat pumps and chillers	EnergyPlus	2022	[30]
AHU	TRNSYS	2020	[25]
AHU	SIMBAD	2020	[29]
AC systems	EnergyPlus, MATLAB, OpenStudio	2020	[28]
AHU	EnergyPlus, OpenModelica	2019	[26]
FCU	MATLAB	2019	[27]
Central water system	N/A	2018	[31]

**Table 2:** Brief summary of related work addressing the knowledge discovery approach in HVAC FDD. For clarity, all acronyms are provided in the Abbreviations list.

Target (sub)System	Technique	Year	Ref.
AC systems	rule based	2021	[35]
AHU	rule based	2021	[33]
AHU	rule based	2021	[32]
AC systems	rule based	2021	[37]
AHU	rule based	2020	[34]
AHU	rule based	2018	[36]

**Table 3:** Brief summary of related work addressing the data-driven approach in HVAC FDD. For clarity, all acronyms are provided in the Abbreviations list.

Target (sub)System	Technique	Year	Ref.
Central water system	Autoencoder, LSTM	2021	[42]
AHU	self-MLP	2021	[43]
Heat pumps and chillers	KPCA	2021	[44]
AHU	Clustering	2021	[45]
Heat pumps and chillers	XGBoost model	2021	[46]
AHU	MCNN	2021	[47]
AHU	unfold-PCA	2021	[48]
AHU	MLP	2021	[49]
Heat pumps and chillers	TCN	2021	[50]
Heat pumps and chillers	DANN	2021	[51]
AHU	TBKSFA	2021	[52]
AHU	SAE	2021	[53]
AC systems	Modified GAN, self-MLP	2021	[54]
Central water system	CART	2021	[55]
Heat pumps and chillers	LR, RF, SVM; MLP, MPC; MOPSO	2021	[56]
AHU	DTO-DRNN	2021	[57]
AHU	CC-RF	2021	[58]
AHU	HRF–SVM	2021	[59]
Heat pumps and chillers	CNN with Grad-CAM	2021	[60]
Heat pumps and chillers	MLP, CART	2020	[61]
AC systems	IF, CF	2020	[62]
Heat pumps and chillers	CWGAN, SVM, MLP	2020	[63]
Heat pumps and chillers	Ensemble (KNN-SVM-RF)	2020	[64]
AHU	Clustering	2020	[65]
AHU	OC-SVM	2020	[66]
AHU	CWGAN, ensemble (SVM-RD-DT)	2020	[67]
AHU	RT	2020	[68]
AHU	DT, Episode-based association	2020	[69]
FCU	Clustering	2020	[70]
HVAC	CPA	2020	[71]
Heat pumps and chillers	CNN	2019	[72]
AHU	Ensemble (WGAN-SVM)	2019	[73]
AHU	HMM	2019	[74]
AHU	DMG	2019	[75]
FCU	LMCSVM	2018	[76]
FCU	MC-SVM	2018	[77]

**Table 4:** Supervised learning used in the research from the selected literature. If the record contains a certain value for Software, then it is actually a representative of a hybrid approach in which some of the data-driven techniques are implemented. For clarity, all acronyms are provided in the Abbreviations list.

Target (sub)System	Algorithm	Synthetic Data	Sample	Software	Year	Ref.
HVAC in transport	BRT	Y		MATLAB	2021	[80]
HVAC in transport	GPR, SVM, RF	Y		MATLAB	2021	[81]
AHU	HRF–SVM	ASHRAE 1312-RP	1 m		2021	[59]
Heat pumps and chillers	CNN with Grad-CAM	ASHRAE 1043-RP	1 m		2021	[60]
Heat pumps and chillers	XGBoost model				2021	[46]
AHU	MCNN	Y		TRNSYS	2021	[47]
AHU	2-D CNN	ASHRAE 1043-RP			2021	[82]
Heat pumps and chillers	TCN				2021	[50]
Heat pumps and chillers	DANN	ASHRAE 1312-RP		TRNSYS	2021	[51]
AHU	MLP	ASHRAE 1312-RP	1 m		2021	[83]
AHU	SAE	Y		EnergyPlus	2021	[53]
Central water system	CART				2021	[55]
Heat pumps and chillers	LR, RF, SVM; MLP; MOPSO				2021	[56]
AHU	RACNN		35 s		2021	[84]
AHU	Bayesian inference				2021	[85]
AHU	DTO-DRNN		1 m		2021	[57]
AHU	CC-RF		35 s		2021	[58]
AHU	DBN				2020	[86]
AHU	ARX, RF			IDA ICE	2020	[87]
Heat pumps and chillers	MLP, CART				2020	[61]
Heat pumps and chillers	Ensemble (KNN-SVM-RF)	ASHRAE 1043-RP	1 m		2020	[64]
FCU	DT		180 s	OpenModelica	2020	[88]
AHU	RT	Y		IDA ICE	2020	[68]
AHU	DT, Episode-based association				2020	[69]
Central water system	Bayesian method	Y	10 m		2020	[89]
AHU	Naive Bayes classifier			EnergyPlus	2020	[90]
AC systems	MSPCA-SVM	Y		TRNSYS	2020	[91]
AC systems	Bayesian classifier				2020	[92]
Heat pumps and chillers	CNN				2019	[72]
AC systems	XGBoost	ASHRAE 1312-RP		EnergyPlus	2019	[93]
Heat pumps and chillers	DBN				2019	[94]
AHU	Ensemble (WGAN-SVM)	ASHRAE 1312-RP	1 m		2019	[73]
AHU	HMM	ASHRAE			2019	[74]
AHU	DMG				2019	[75]
FCU	MC-SVM	ASHRAE			2018	[77]

**Table 5:** Unsupervised learning used in the research from the selected literature. For clarity, all acronyms are provided in the Abbreviations list.

Target (sub)System	Algorithm	Synthetic Data	Sample	Software	Year	Ref.
Central water system	Autoencoder, LSTM				2021	[42]
AHU	Clustering				2021	[45]
AHU	unfold-PCA		15 m		2021	[48]
HVAC in transport	Fuzzy-Based RCM				2021	[99]
Heat pumps and chillers	hierarchy fuzzy post mining		10 m		2021	[96]
AHU	TBKSFA	ASHRAE 1312-RP	1 m		2021	[52]
AHU	Clustering		1 h		2020	[65]
AC systems	IF, CF				2020	[62]
AHU	CWGAN, ensemble (SVM-RD-DT)	ASHRAE 1312-RP	1 m		2020	[67]
AHU	SSIM	Y			2020	[100]
AHU	PCA	Y		HVACISM+	2020	[101]
FCU	Clustering				2020	[70]
HVAC	CPA				2020	[71]
Heat pumps and chillers	ARM FP-growth				2019	[97]

**Table 6:** Semi-supervised learning used in the research from the selected literature. For clarity, all acronyms are provided in the Abbreviations list.

Target Sub(System)	Algorithm	Synthetic Data	Sample	Software	Year	Ref.
AHU	LSTM-SVDD	Y		OpenModelica	2022	[103]
AHU	self-MLP				2021	[43]
Heat pumps and chillers	KPCA	ASHRAE 1043-RP			2021	[44]
AHU	MLP	ASHRAE 1312-RP			2021	[49]
AC systems	Modified GAN, self-MLP	ASHRAE 1043-RP			2021	[54]
Heat pumps and chillers	Clustering, ARM, DT	ASHRAE 1312-RP			2021	[95]
Heat pumps and chillers	CWGAN, SVM, MLP	ASHRAE 1043-RP			2020	[63]
AHU	TARM, CART				2020	[98]
AHU	OC-SVM				2020	[66]
HVAC	MSIPCA-SVM	Y	1 h	TRNSYS	2020	[102]
FCU	LMCSVM		15 m		2018	[76]

**Table 7:** Brief summary of related work addressing the hybrid approach in HVAC FDD. For clarity, all acronyms are provided in the Abbreviations list.

Target (sub)System	Technique	Software	Year	Ref.
AHU	LSTM-SVDD	OpenModelica	2022	[103]
HVAC in transport	BRT	MATLAB	2021	[80]
HVAC in transport	GPR, SVM, RF	MATLAB	2021	[81]
AHU	2-D CNN	TRNSYS	2021	[82]
AHU	MLP	TRNSYS	2021	[83]
AHU	RACNN	N/A	2021	[84]
AC systems	Kalman filter	EnergyPlus	2021	[104]
AHU	Bayesian inference	N/A	2021	[85]
Heat pumps and chillers	Clustering, ARM, DT	N/A	2021	[95]
Heat pumps and chillers	ARM FP-growth	N/A	2019	[96]
HVAC in transport	Fuzzy-Based RCM	N/A	2021	[99]
AHU	LSM	N/A	2020	[106]
HVAC	MSIPCA	TRNSYS	2020	[102]
AC systems	MSPCA, SVM	TRNSYS	2020	[91]
AC systems	Bayesian classifier	N/A	2020	[92]
AHU	Naive Bayes classifier	EnergyPlus	2020	[90]
AHU	PCA	HVACSIM+	2020	[101]
Heat pumps and chillers	Multi agent system	N/A	2020	[105]
AHU	ARX, RF	IDA ICE	2020	[87]
FCU	DT	OpenModelica	2020	[88]
AHU	SSIM	N/A	2020	[100]
AHU	TARM, CART	N/A	2020	[98]
AHU	DBN	N/A	2020	[86]
Central water system	Bayesian method	N/A	2020	[89]
AC systems	XGBoost	EnergyPlus	2019	[93]
Heat pumps and chillers	DBN	N/A	2019	[94]
Heat pumps and chillers	hierarchy fuzzy post mining	N/A	2021	[97]

**Table 8:** A summarized comparison of described ML models in the field of FDD for HVAC systems. While the first five listed models refer to supervised learning, the last two refer to unsupervised learning. For clarity, all acronyms are provided in the Abbreviations list.

Model	Advantages	Disadvantages
SVM	– Ability to seamlessly expand the feature space through the use of kernels. – Modeling (non-linear) distributions with relatively high accuracy. – Overall good performance due to maximum-margin separation.	– Requires longer training times for learning from larger datasets. – Low interpretability.
ANN	– Capable of learning complex spatially- and/or temporally-dependent fault nature. – Capable of simultaneously learning both feature extraction and the objective in an end-to-end optimization process (i.e., deep learning). – Ability to diagnose multiple faults simultaneously. – Model learning can be parallelized.	– Performance highly dependent on network architecture, which is influenced by numerous hyperparameters that need to be tuned manually. – Deep architectures usually take longer to train. – Low interpretability.
DT	– Ability to model highly complex representations, through input space partitioning. – Can achieve excellent performance. – Highly interpretable.	– Model performance may not be guaranteed in complex multi-class faults. – Susceptible to overfitting.
RF	– The best trade-off between model complexity and generalization ability. – Usually is more accurate than DTs. – Robust to outliers. – Model learning can be parallelized.	– High computational complexity due to ensembling. – Low interpretability.
XGBoost	– Efficient with large datasets. – Insensitive to distribution skewness.	– Difficult to tune. – Performs poorly on sparse and unstructured data. – Low interpretability.
Clustering	– Ability to discover patterns among the data points. – Can extract useful information from intra-cluster relationships.	– More sensitive to data quality. – Similarities can be difficult to find in practice, especially when classifying multiple faults. – New faults that are not recognized as a member of created groups cannot be diagnosed properly. – Low interpretability.
ARM	– Ability to identify strong rules in the data. – Tool for finding inherent regularities in the data. – Highly interpretable. – Rule learning can be parallelized.	– Usually requires categorical variables as input. – The results often consist of a large amount of redundancy, which requires post-mining steps. – The complex connections between multiple features can be challenging to interpret in comparison to supervised methods.

## Data Availability

Not applicable.

## Supplementary Materials

The following are available online at https://www.mdpi.com/article/10.3390/s23010001/s1, Table S1: Sources used in SLR.

## Abbreviations

The following abbreviations are used in this manuscript:

AC	Air Conditioning Systems
ACODAT	Autonomous Cycle of Data Analysis
AHU	Air Handling Units
ANN	Artificial Neural Network
ARM	Association Rule Mining
ARX	autoregressive
ASHRAE	American Society of Heating, Refrigerating and Air-Conditioning Engineers
BEMS	Building Energy Management System
BPNN	Back Propagation Neural Network
BRT	Boosted Regression Tree
BT	Binary Tree
CART	Classification and Regression Tree
CC-RF	Classifier Chains-Random Forest
CF	Cascade Forest
CNN	Convolutional Neural Network
CPA	Change Point Analysis
CWGAN	Conditional Wasserstein Generative Adversarial Network
DAG	Directed Acyclic Graph
DANN	Domain Adversarial Neural Network
DBN	Diagnostic Bayesian Network
DL	Deep Learning
DMG	Diagnostic Multi-Query Graph
DRNN	Deep Recurrent Neural Networks
DT	Decision Tree
DTO-DRNN	Deep Transition Output-Deep Recurrent Neural Networks
FCU	Fan Coil Units
FDD	Fault Detection and Diagnosis
FP	Frequent Pattern
GAN	Generative Adversarial Network
GMM	Gaussian Mixture Model
GPR	Gaussian Process Regression
Grad-CAM	Gradient-weighted Class Activation Mapping
HMM	Hidden Markov Model
HRF	Hybrid Random Forest
HVAC	Heating, Ventilation, and Air Conditioning
IEA	International Energy Agency
IF	Isolation Forest
Isomap	Isometric Feature Mapping
KNN	K-Nearest Neighbors
KPCA	Kernel Principal Component Analysis
LLE	Locally Linear Embedding
LMC-SVM	Linear Linear Multiclass Support Vector Machine
LR	Logistic Regression
LSM	Least-Squares Method
LSTM-SVDD	Long Short-Term Memory
MCNN	Multiscale Convolutional Neural Networks
MC-SVM	Multiclass Support Vector Machine
ML	Machine Learning
MLP	Multi-Layer Perceptron
MOC-RCE	Multi-Objective Clustering-Rapid Centroid Estimation
MOPSO	Multiobjective Particle Swarm Optimization
MPC	Model Predictive Control
MSIPCA	Multiscale Interval Principal Component Analysis
MSPCA	Multiscale PCA
OAA	One-Against-All
OAO	One-Against-One
OC-SVM	One-class Support Vector Machine
PCA	Principal Component Analysis
RACNN	Rule and Convolutional Neural Networks
RACNN	Rule-based method and Convolutional Neural Network
RCM	Reliability-Centered Maintenance
RCU	Refrigeration Compressor Rack Unit
RF	Random Forests
RNN	Recurrent Neural Networks
RTU	Rooftop Units
SAE	Supervised Auto-Encoder
SFA	Slow Feature Analysis
SLR	Systematic Literature Review
SPE	Square Prediction Error Statistics
SSIM	Structural Similarity Index Measure
SVDD	Support Vector Data Description
SVM	Support Vector Machine
SVR	Support Vector Regression
TARM	Temporal Association Rules Mining
TBKSFA	Three-way Data Based Kernel Slow Feature Analysis
TCN	Temporal Convolutional Network
t-SNE	t-distributed Stochastic Neighbor Embedding
VRF/VRV	Variable Refrigerant Flow / Volume Systems
WGAN	Wasserstein Generative Adversarial Network
XGBoost	Extreme Gradient Boosting
